# Therapeutic response of oral chronic graft-versus-host disease to topical corticosteroids according to the 2014 National Institutes of Health (USA) consensus criteria

**DOI:** 10.4317/medoral.26203

**Published:** 2023-10-12

**Authors:** Wachiravit Siripornkitti, Nawaporn Pengpis, Chantiya Chanswangphuwana, Titipong Prueksrisakul

**Affiliations:** 1 Postgraduate student. Oral Medicine Department, Faculty of Dentistry, Chulalongkorn University, Bangkok, Thailand; 2 Division of Oral Diagnostic Science, Faculty of Dentistry, Thammasat University, Pathum Thani, Thailand; 3 Division of Hematology, Department of Medicine, Faculty of Medicine, Chulalongkorn University and King Chulalongkorn Memorial Hospital, Bangkok, Thailand; 4 Center of Excellence in Translational Hematology, Faculty of Medicine, Chulalongkorn University and King Chulalongkorn Memorial Hospital, Bangkok, Thailand; 5 Assistant Professor. Oral Medicine Department, Faculty of Dentistry, Chulalongkorn University, Bangkok, Thailand

## Abstract

**Background:**

Chronic graft-versus-host-disease (cGVHD) is a major cause of morbidity and mortality after allogeneic hematopoietic stem cell transplantation. The oral cavity is one of the most frequently affected anatomic sites and is affected in 70% of all patients who develop cGVHD. The objective of this study was to determine the therapeutic response to topical corticosteroids and clinical outcome of patients with oral cGVHD using the 2014 NIH consensus criteria.

**Material and Methods:**

The oral manifestations of cGVHD were collected at the first and the follow-up (FU) visits after the therapeutic treatment of oral GVHD. The FU intervals were: FU0, first visit; FU1, 0-1 month; FU2, 1-3 months; FU3, 3-6 months; FU4, 6-9 months; and FU5, 9-12 months. The oral cGVHD activity was assessed using the NIH modification of the Schubert Oral Mucosa Rating Scale (OMRS) and Thongprasom sign score. The functional impact was assessed by the organ-specific severity score.

**Results:**

Fourteen patients (93.3%) at FU0 were being treated with at least one form of systemic immunosuppressive therapy, i.e., prednisolone, cyclosporin, and tacrolimus. The OMRS was reduced between FU0 and FU3 (*p* < 0.001), FU0 and FU4 (*p* < 0.001), and FU0 and FU5 (*p* = 0.004). The organ-specific severity scores were also reduced between FU0 and FU4 (*p* = 0.016), and FU0 and FU5 (*p* = 0.001). There was no significant difference in the highest Thongprasom sign score between all follow-up intervals (FU0-FU5) (*p* = 0.201). One patient (6.7%) at FU4 and three patients (20.0%) at FU5 did not receive topical corticosteroid therapy for oral cGVHD.

**Conclusions:**

The oral cGVHD lesions and functional impacts improved within 6 months and 9 months, respectively. However, most of the patients required topical corticosteroid therapy for more than 1 year to control their symptoms and lesions.

** Key words:**Oral chronic graft-versus-host disease, 2014 National Institutes of Health consensus criteria, Hematopoietic cell transplantation, therapeutic response, topical corticosteroid.

## Introduction

Chronic graft-versus-host-disease (cGVHD) is a major cause of morbidity and mortality after allogeneic hematopoietic stem cell transplantation (allo-HSCT), affecting up to 70% of recipients and commonly involve the skin, mouth, eyes, gastrointestinal tract, liver, lungs, and joints (1,2). The clinical features of cGVHD resemble several autoimmune or immune-mediated conditions, such as systemic lupus erythematosus, scleroderma, and lichen planus that are characterized by fibrosis and chronic inflammation (3).

The oral cavity is one of the most frequently affected anatomic sites and is affected in 70% of all patients who develop cGVHD. The oral mucosa and salivary glands can be affected, causing pain and discomfort, limited oral intake, dysfunction, difficulty in chewing and swallowing, and an increased risk of dental caries and other oral infections (4,5). The clinical presentation of several autoimmune conditions is similar to that of oral cGVHD. Lichen planus presents as an oral mucosal disease with a series of changes similar to those seen in oral mucosal cGVHD. Sjögren’s syndrome presents as salivary gland dysfunction, similar to the dry mouth seen in salivary gland cGVHD, however, the pathology is different. Scleroderma is associated with limited mouth movement as part of its systemic sclerosis pattern of cGVHD (6). Oral cGVHD has a negative effect on oral functional capacity, oral health, and quality of life of the patient. Oral cGVHD patients also experience decreased oral health-related quality of life, taste alteration, and increased levels of oral-related pain and dry mouth (7).

The goals of oral cGVHD therapy are to reduce the symptoms, painful lesions, and preventing and managing secondary complications. cGVHD patients that have multiple organ involvement require systemic treatment with corticosteroids or other immunomodulatory agents. Topical management of oral cGVHD may be used as an adjunctive therapy to systemic treatment in locally refractory cases or as the lone therapy in cases where the oral cavity is the only site involved (8). The first-line therapy for oral mucosal GVHD is intensive topical corticosteroid therapy that can be delivered in various formulations (9,10).

The National Institutes of Health (NIH) established consensus criteria for diagnosing cGVHD and tools for scoring and assessing the severity of cGVHD in 2005 (11). In 2014, the NIH consensus was revised with updated recommendations for the interpretation and assessment of organs and overall responses to cGVHD treatment. The major changes included the removal of several clinical parameters of treatment outcomes, and updates of new organ scales to assess the clinical response (12). However, there are few studies that have evaluated the therapeutic response to topical corticosteroids and clinical outcome of patients with oral cGVHD. The objective of this study was to determine the therapeutic response and clinical outcome of patients with oral cGVHD.

## Material and Methods

Patients who had undergone allogeneic hematopoietic cell transplantation at the Hematology Unit at King Chulalongkorn Memorial Hospital and were diagnosed with oral cGVHD and received treatment at the Oral Medicine clinic at the Faculty of Dentistry, Chulalongkorn University, were enrolled in this retrospective study. The inclusion criteria were patients with a diagnosis of oral cGVHD according to the 2014 NIH diagnostic criteria and received topical corticosteroid therapy at the Oral Medicine clinic, Faculty of Dentistry, Chulalongkorn University. Only patients who were referred by physicians from the Hematology Unit at King Chulalongkorn Memorial Hospital were included. The exclusion criteria were patients with a diagnosis of oral acute GVHD (aGVHD) based on the 2014 NIH diagnostic criteria. The patient’s medical data comprising demographic data, patient characteristics, and oral manifestations were collected and evaluated from 2010-2022. The physicians at the Hematology Unit diagnosed the patients as having a cGVHD condition, whereas the dentists at the Oral Medicine clinic diagnosed the patients as having an oral cGVHD condition according to the 2014 NIH consensus criteria (13).

This study was approved by The Human Research Ethics Committee of the Faculty of Dentistry, Chulalongkorn University (HREC-DCU 2022-022), and the Institutional Review Board of the Faculty of Medicine, Chulalongkorn University (COA No. 1176/2022).

The sample size was calculated using n4studies with proportion (p) = 0.80, error (d) = 0.2, and alpha (α) = 0.05 according to the prevalence of oral cGVHD from Fassil *et al*. (7). The estimated sample size was 16.

The oral cGVHD activity was assessed with the NIH modification of the Schubert Oral Mucosa Rating Scale (OMRS), which has an oral mucosal score of 0-12. Three oral cGVHD manifestations were assessed with the NIH Mouth cGVHD Activity Assessment Scale: a) mucosal erythema (color intensity and percent oral surface area); b) lichen-like changes (percent oral surface area); and c) ulcerations (percent oral surface area) (Table 1) (12).

The Thongprasom sign score was used to determine the severity and extension of the lichen planus-like changes at the involved locations with scores ranging from 0-5, score 0 for no lesion or normal mucosa; score 1 for mild white striae with no erythematous area; score 2 for white striae with an atrophic area less than 1 cm2; score 3 for white striae with an atrophic area more than or equal to 1 cm2; score 4 for white striae with an erosive area less than 1 cm2; and score 5 for white striae with an erosive area more than or equal to 1 cm2 (14).

The organ-specific severity scoring system according to the 2014 NIH consensus criteria was used to assess the severity and extension of oral cGVHD on a scale of 0-3 based on clinical presentation and functional impact. The severity of involvement was a 4-point scale, score 0 for no symptoms; score 1 for mild symptoms with disease signs, but not markedly limiting oral intake; score 2 for moderate symptoms with disease signs with partial limitation of oral intake; and score 3 for severe symptoms with disease signs on examination with major limitation of oral intake (13).

According to the NIH recommendations, the therapeutic response measurements should be made at regular intervals, e.g., every 3 months. The manifestations of oral cGVHD at 2 time points must be compared to assess the therapeutic response (12). The oral manifestations of cGVHD and organ-specific severity scores were collected and assessed from the clinical charts of the first and the follow-up (FU) visits after treatment of oral cGVHD. The FU intervals comprised FU0, first visit; FU1, 0-1 month; FU2, 1-3 months; FU3, 3-6 months; FU4, 6-9 months; and FU5, 9-12 months.

**Table 1:**
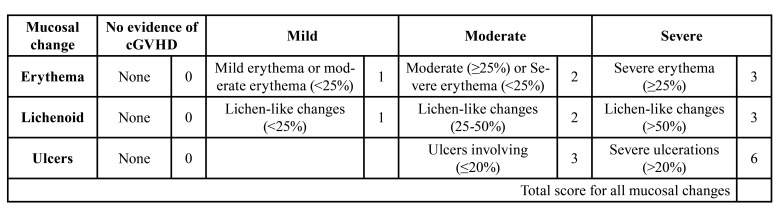
Oral Mucosa Rating Scale assessed with the NIH Mouth cGVHD Activity Assessment Scale.

The statistical analyses were performed using SPSS version 28 at a significance level of 0.05. Descriptive statistics were used to present the demographic data of the cGVHD patients. The differences in the OMRS, the highest Thongprasom sign score, and the organ-specific severity score of oral cGVHD among the follow-up intervals (FU0-FU5) were analyzed by the Friedman test and Dunn’s post hoc test.

## Results

Fifteen patients with oral cGVHD who received therapeutic therapy with topical corticosteroids for 9-12 months were included in this retrospective study to evaluate their therapeutic response. The mean patient age at the first visit (FU0) was 41.3 ± 7.9 years old (range 26-55 years old). The median number of days from transplant to oral cGVHD diagnosis was 255 (range 45-867 days). Fourteen patients (93.3%) at FU0 were being treated with at least one form of systemic immunosuppressive therapy, i.e., prednisolone (*n*=7, 46.7%), cyclosporin (*n*=9, 60.0%), and tacrolimus (*n*=5, 33.3%). Thirteen patients still required systemic immunosuppressive therapy at FU5. The demographic data and characteristics of the oral cGVHD patients are summarized in Table 2.

**Figure 1:**
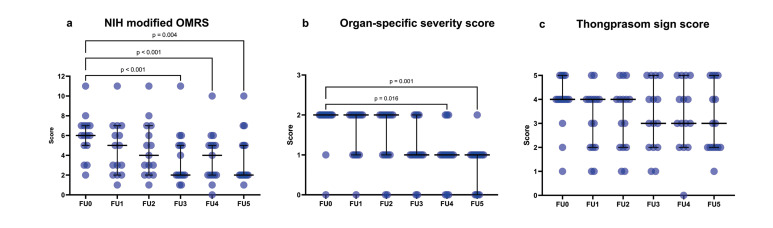
Dot plot with median and interquartile range differences the NIH modified OMRS scores. (a), Organ-specific severity score (b), and highest Thongprasom sign score (c) among the follow-up intervals (FU0-FU5).

The OMRS, highest Thongprasom sign score, and organ-specific severity score of the patients were compared among the follow-up (FU) intervals: FU0, FU1, FU2, FU3, FU4, and FU5. Fig. 1 presents the median, interquartile range, and statistical analyses of the differences in the OMRS score, organ-specific severity score, and highest Thongprasom sign score among the follow-up intervals. The statistical analyses demonstrated that there were significant differences in the OMRS among all follow-up intervals (FU0-FU5) (*p* < 0.001). Dunn’s post hoc test indicated that the OMRS was significantly reduced between FU0 and FU3 (*p* < 0.001), FU0 and FU4 (*p* < 0.001), and FU0 and FU5 (*p* = 0.004). Furthermore, the median OMRS was reduced from 6 at FU0 to 2 at FU5.

There were significant differences in the organ-specific severity score among all follow-up intervals (FU0-FU5) (*p* < 0.001), with significant reductions between FU0 and FU4 (*p* = 0.016), and FU0 and FU5 (*p* = 0.001).

**Table 2:**
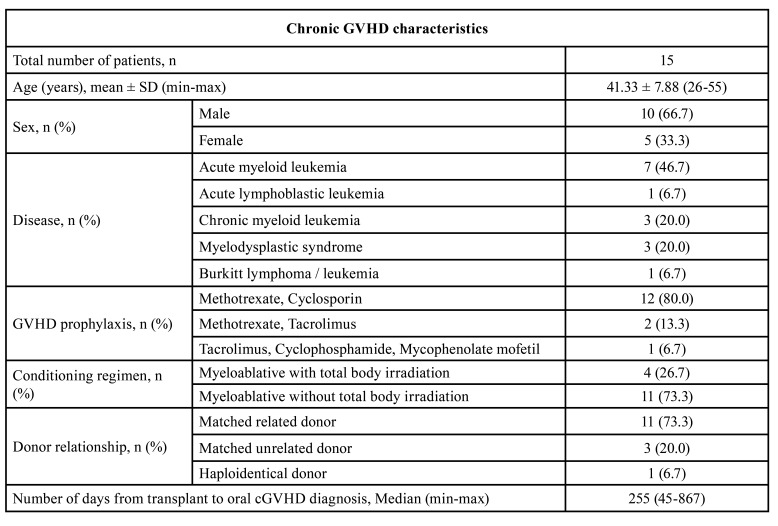
Demographic data and chronic GVHD characteristics.

At FU0, thirteen patients (86.7%) had a score 2, one patient had a score 1, and one patient had a score 0. Therefore, the median organ-specific severity score at FU0 was score 2 and was reduced to score 1 at FU5.

**Figure 2:**
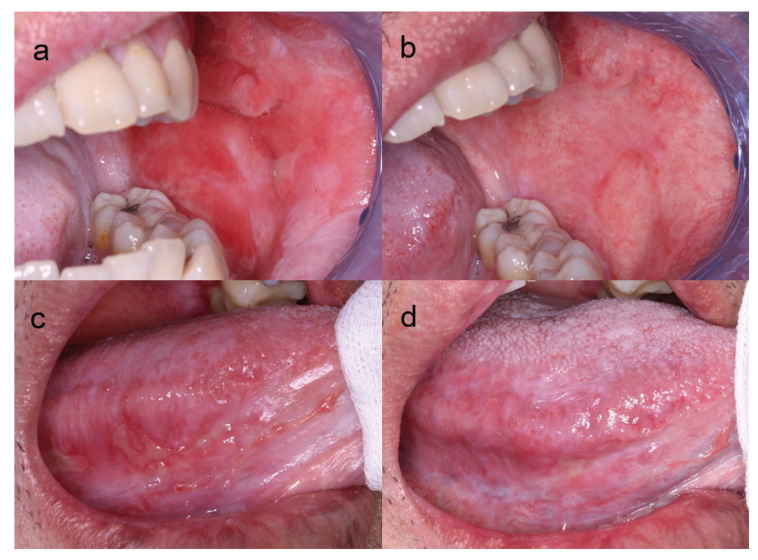
Left buccal mucosa of a patient (ID 10) at FU0 (a) with erythema and reticular areas and FU5 (b) with reticular and mild erythema areas, right lateral tongue of a patient (ID 12) at FU0 (c) with erythema areas, reticular areas and ulcerations and FU3 (d) with reticular areas.

There were no significant differences in the highest Thongprasom sign score among all follow-up intervals (FU0-FU5) (*p* = 0.201). The median of the highest Thongprasom sign score was reduced from 4 at FU0 to 3 at FU5. The median and interquartile range of the OMRS, organ-specific severity score, and highest Thongprasom sign score among FU0-FU5 are presented in Table 3. Representative lesions at the left buccal mucosa and right lateral tongue of patients with oral cGVHD are presented in Fig. 2.

**Figure 3:**
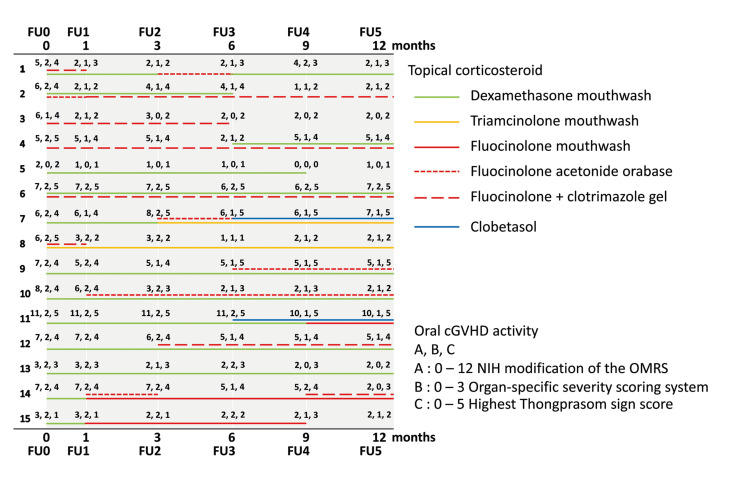
Summary of the therapeutic response of oral cGVHD patients per follow-up interval.

The topical corticosteroid therapies and therapeutic responses during the follow-up (FU) intervals are summarized in Fig. 3. At the 1 month follow-up (FU1), all fifteen patients received at least one topical corticosteroid therapy consisting of dexamethasone mouthwash (*n*=9, 60.0%), fluocinolone + clotrimazole gel (*n*=2, 13.3%), and topical corticosteroid combinations (*n*=4, 26.7%). All fifteen patients still required at least one topical corticosteroid therapy during the 1-3 month follow-up (FU2) and 3-6 month follow-up (FU3) period. One patient (6.7%) at FU4 and three patients (20.0%) at FU5 did not receive topical corticosteroid therapy because there were no symptoms in their oral cavity and their oral cGVHD activity was minimal. During the therapeutic therapy for oral cGVHD, eight patients (53.3%) developed oral candidiasis. These patients received antifungal treatment (nystatin suspension) so they could continue their topical corticosteroid regimen. Four patients (26.7%) reported they had limited mouth opening that decreased their maximum mouth opening. Three patients (20.0%) also reported that they had xerostomia symptoms that caused oral discomfort and dysphagia.

**Table 3:**
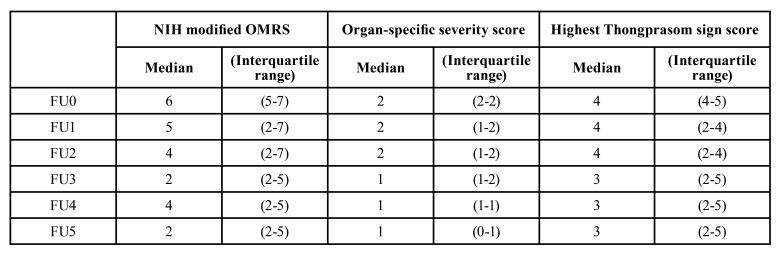
The median and interquartile range of the NIH modified OMRS, organ-specific severity score, and highest Thongprasom sign score among FU0-FU5.

## Discussion

Chronic GVHD is a complication of allo-HSCT (4,7,11). Oral cGVHD causes significant pain and discomfort, dysfunction, and limits oral intake (5,15). The most common symptom of oral cGVHD patients is sensitivity to spicy, acidic, and strongly flavored food (4,16). The organ-specific severity scores in this study indicated that most of the patients in this study had symptomatic oral cGVHD with a median of score 2 at FU0. These symptoms have a negative impact on the oral health, functional capacity, and quality of life of affected patients (7).

The goals of oral cGVHD therapy are reducing the symptoms and painful lesions, and preventing and managing secondary complications (9,17). Pengpis N *et al*. found that the most common oral manifestation of cGVHD was a white reticular area with an erosive area (18). Although systemic treatment is beneficial for oral cGVHD, additional local immunosuppressive therapy (e.g., topical corticosteroids and topical tacrolimus) is required in some patients (10,19). Although most of the patients in this study were being treated with systemic immunosuppressive therapy at FU0, the patients also required topical corticosteroid therapy for managing their oral cGVHD due to the severity of their oral lesions and symptoms. In the locally refractory cases in this study, the patients received a combined topical corticosteroid regimen for additional management based on their symptoms and manifestations. Similar to the systemic management of cGVHD, the management regimens for oral cGVHD are dynamic. The therapeutic response of each patient can be affected by patient compliance with topical corticosteroid therapy. In the present study, some patients were not fully compliant with their assigned treatment regimens, which might have affected the results. The systemic immunosuppressive therapy that these patients received could also be a factor that affected the therapeutic response of oral cGVHD in this study.

The therapeutic response measurements performed according to the NIH recommendations for the follow-up intervals demonstrated significant reductions in the OMRS between FU0 and FU3, FU0 and FU4, and FU0 and FU5. The median of the OMRS was reduced from 6 at FU0 to 2 at FU5. These results are similar to those of Shazib MA *et al*. in 2020, who reported that there was an overall reduction in the median oral mucosal score from 3 at the 1-month follow-up to 1 at the 24-month follow-up for all evaluated subjects (2). We also found that the organ-specific severity scores were significantly reduced between FU0 and FU4, and FU0 and FU5. These results corresponded to the decrease or discontinuation of topical corticosteroid therapy in some patients at FU4 and FU5.

The Thongprasom sign score was used to determine the severity and extension of the lichen planus-like changes in this study. The results revealed that there were no significant differences in the highest Thongprasom sign score between all follow-up intervals (FU0-FU5). Because there were several oral mucosa sites where the patients developed lichen planus-like changes, the highest Thongprasom sign score does not represent the overall oral lichen planus-like changes or overall oral manifestations. However, the highest Thongprasom sign score can represent the most severe oral cGVHD lesion of the patients. At FU5, several patients still had a high Thongprasom sign score that corresponded to the continuation of topical corticosteroid therapy in these patients.

There are several limitations in this study. First, the included patients in this study were referred by physicians for the management of oral cGVHD when systemic immunosuppressive therapy alone could not control their oral cGVHD symptoms and manifestations. Therefore, the results of our study may not represent the therapeutic response of the patients with mild symptoms or asymptomatic oral cGVHD. In addition, the study had a retrospective design that has multiple limitations, including missing data, recorder bias, and selection bias. The missing data are pain score, maximum mouth opening measurement, and other treatment records. Lastly, the small sample size (*n*=15) is a limitation for data analysis. Future studies are needed with larger sample sizes including patients with mild symptoms, asymptomatic, and symptomatic oral cGVHD and a prospective study design would be better to gather more data for a long-term follow-up study of oral cGVHD treatment.

## Conclusions

This 1-year follow-up study demonstrated that topical corticosteroid therapy improved the oral cGVHD lesions in patients within 6 months that resulted in a decreased NIH modification of OMRS. Moreover, the functional impacts of the patients were improved within 9 months that reflected the decreased organ-specific severity score after topical corticosteroid therapy. However, most of the patients required topical corticosteroid therapy for more than 1 year to control their symptoms and lesions.
